# Glances at the Hospitals

**Published:** 1903-10-03

**Authors:** 


### GLANCES AT THE HOSPITALS.

### BRISTOL GENERAL HOSPITAL.

The ordinary receipts amounted to £10,628, and the ordinary expenditure to £12,468, leaving an adverse balance of £1,840. The workpeople's subscriptions maintain a satisfactory increase, and now reach the respectable figure of £2,050. Lady Smyth, of Ashton Court, sent a donation of £1,500, and Mr. W. Proctor and Mr. Sparks Evans each sent £500. The committee recognise the necessity of keeping the institution in line with the latest requirements of science and medicine, and they find that this course entails a heavy expenditure at times. Satisfactory results have been obtained by the Finsen-light treatment of lupus, and in the bacteriological department 360 specimens have been examined. At the beginning of the year the hospital contained 164 patients, and 2,290 were admitted since. Of this total 1,411 were discharged cured, 512 relieved, 133 for various reasons, 234 died, and 164 remained under treatment. An analysis of the more important medical and surgical cases is given. There were 31 cases of pleurisy with 1 death, 14 cases of pneumonia with 5 deaths. This seems a high mortality, and it would be interesting to have a column for remarks on some of these cases. There were 9 cases of appendicitis, all of which recovered. There were 12 cases of strangulated hernia with 1 death, and the operation for the radical cure of hernia was performed 50 times, all being successful. The surgical operations are not placed separately, as they usually are in hospital reports, and there is no anæsthetic table.

### CLACKMANNAN COUNTY HOSPITAL.

The income amounted to £1,316 and the expenditure to £1,276, showing a balance of £40 on the right side; and it is no wonder that the committee have pleasure in reporting an increase in the subscriptions, donations, and collections. The average number of beds occupied was 18, and the average cost per bed was £59 15s. A complete list of all the patients treated during the year is published, and this table shows the initials, sex, age, previous residence, occupation, days in hospital, disease, and the result. It is therefore possible to learn the result of treatment in every case, therefore no fault should be found with the statistics thus given; but for the reader who wishes to compare one hospital report with another, the usual tables are better and easier to consult. The hospital contained 19 patients at the beginning of the year, and there were 169 admissions during it. Of the total, 87 were discharged recovered, 75 relieved, 4 not relieved, and 13 died. There were 9 under treatment on the date of this report.

### LEICESTER INFIRMARY.

The year began with an adverse balance of £1,108 and it ended with one of £4,150, showing that the year's income was insufficient to meet the year's expenditure by more than £3,000, and, as the committee point out, the benefits of the institution must be curtailed unless the finances can be placed on a more satisfactory footing. It is worthy of record that some of the subscribers have doubled or quadrupled their subscriptions, while the workpeople have increased their donations by £338 and that they contributed a total of £4,086 during the year. The average daily number of in-patients was 188, the average stay in the hospital was 24 days, the average cost of each in-patient was £4 4s. 7d., and the average cost per week per patient was £1 5s. 9d. On January 1st the infirmary contained 153 patients, and 2,155 were admitted during the year. Of the total 1,549 were discharged either cured or relieved, 460 for various reasons, 158 died, and 141 remained under treatment. In the Children's Hospital there was a total of 576, and of these 364 were discharged cured or relieved, 120 for various reasons, 53 died, and 39 remained under treatment. Taking the Infirmary and Children's Hospital together, there was an average of 188 patients in residence daily. The medical and surgical tables are most unsatisfactory, as no results are given anywhere, except in the $x$-ray department. Here we learn there were 223 patients treated or examined. In the former there were 14 cases of lupus and 8 of cancer. Of these 4 cases of lupus and 3 of cancer have been completely cured as far as can be judged at present; while the remainder have been benefited in various ways, excepting 3 or 4, who received no relief. Of course the cancer cases were of the skin or superficial tissues, but nevertheless the results are most encouraging. Anæsthetics were administered 2,716 times, but of their nature we hear nothing.

### ESSEX AND COLCHESTER HOSPITAL.

The receipts, including legacies, amounted to £4,399, and the expenditure to £3,380. The income exceeded that of the previous year and the expenditure was rather less, so that the governors were able to wipe off £300 of their debt. It is, however, pointed out that an anonymous donor sent £500, and that a bazaar, organised by Mr. Nash, realised a net profit of £1,636. On January 1st the hospital contained 55 patients, and 750 were admitted during the year, making a total of 805 under treatment. Of these 333 were cured, 300 greatly relieved, 39 relieved, 35 were discharged for various reasons, 37 died, and 59 remained in the wards. The usual medical and surgical tables are not supplied, but there is a table of the operations performed and the results thereof. We note that chloroform was given on 152 occasions, gas and ether on 32, chloroform and ether on 7, and the A.C.E. mixture on 111. It is to be hoped that the next report will contain medical and surgical tables so arranged as to show the results of treatment of the various diseases.

